# Environmental Health Impacts of Concentrated Animal Feeding Operations: Anticipating Hazards—Searching for Solutions

**DOI:** 10.1289/ehp.8831

**Published:** 2006-11-14

**Authors:** Peter S. Thorne

**Affiliations:** College of Public Health, The University of Iowa, Iowa City, Iowa, USA

**Keywords:** air quality, animal confinements, antibiotic resistance, antimicrobial growth promotants, avian influenza, bioaerosols, livestock, poultry, swine, water quality

## Abstract

A scientific conference and workshop was held March 2004 in Iowa City, Iowa, that brought together environmental scientists from North America and Europe to address major environmental health issues associated with concentrated animal feeding operations (CAFOs) in large, industrialized livestock production facilities. After one and a half days of plenary sessions, five expert workgroups convened to consider the most relevant research areas, including respiratory health effects, modeling and monitoring of air toxics, water quality issues, influenza pandemics and antibiotic resistance, and community health and socioeconomic issues. The workgroup reports that follow outline the state of the science and public health concerns relating to livestock production as they apply to each workgroup topic. The reports also identify areas in which further research is needed and suggest opportunities to translate science to policy initiatives that would effect improvements in public and environmental health. Viable solutions to some of the current environmental health problems associated with CAFOs are outlined. In addition, these reports bring to light several major concerns, including air and water contamination, the rise of antibiotic-resistant bacteria in livestock, and the specter of influenza outbreaks arising from siting industrialized poultry and swine production in proximity to each other and to humans.

Dramatic changes in livestock production have occurred over the past two decades. The trend in swine, poultry, and cattle operations has been toward fewer but increasingly larger operations. Traditional crop–livestock farms were balanced in that livestock manure supplied nutrients to grow the crops to feed those livestock. Farmers raised the quantity of livestock their croplands could support. Industrialized livestock production requires drawing feed from a wide area, often far away, whereas manure is distributed to a small, local landmass resulting in soil accumulation and runoff of phosphorus, nitrogen, and other pollutants (Iowa State University and University of Iowa Study Group 2002). The consolidation of the livestock industry has been observed throughout North America and Europe and has led to calls for increased regulation to reduce and control the wastes. The state of Iowa, which produces one-fourth of U.S. pork, exemplifies this trend. The number of farms in Iowa raising hogs decreased from 64,000 in 1980 to 10,500 in 2000—an 84% decrease—while the average number of hogs per farm increased from 250 to 1,430 over this same period (Otto and Lawrence 2000). Farms with more than 500 hogs now account for 65% of the statewide inventory and 75% of the U.S. inventory.

The results of the increasing intensity of livestock operations have been regionally higher levels of air contaminants and increased problems with contamination of surface waters with animal waste. Management practices such as feeding animals with antimicrobial growth promotants and housing poultry and swine in proximity are additional concerns. Fears of the communities and neighbors concerning potential adverse human health effects have increased, leading to the formation of citizen action groups in many locales. These groups have lobbied government officials at the local and regional levels to promulgate and enforce regulations to reduce environmental impacts and health hazards from nearby concentrated animal feeding operations (CAFO). A town meeting sponsored by the National Institute of Environmental Health Sciences (Research Triangle Park, NC) and the University of Iowa, Environmental Health Sciences Research Center (EHSRC), was held in Des Moines, Iowa, in 2001 to bring stakeholders together to seek common ground. This town meeting gave producers, concerned citizens, and regulators the opportunity to discuss the issues. Many areas of discord were identified, and a need for better translation of science to policy was recognized.

Findings from the 2001 town meeting prompted the EHSRC to organize the scientific conference and workshop “Environmental Health Impacts of Concentrated Animal Feeding Operations: Anticipating Hazards—Searching for Solutions” held March 2004 in Iowa City, Iowa, which brought together experts in environmental science from the United States, Canada, Sweden, Denmark, and the Netherlands to address major environmental health issues associated with CAFOs. The conference audience comprised scientists, agriculturalists, producer group representatives, environmental and community activists, government officials, and rural residents. Five workgroups of scientists convened to consider further the major topics and identify the state of the science. Their reports make up this mini-monograph. These reports outline the scientific issues and public health concerns relating to livestock production as it applies to each workgroup topic and identify areas in which further research is needed. They also suggest opportunities to translate science to policy initiatives that would advance public and environmental health.

## Summary of Workshop Recommendations

The Workgroup on Health Effects of Airborne Exposures from CAFOs found a lack of data on the health effects of odors and complex mixtures emanating from CAFOs (Heederik et al. 2006). They also identified a need for research on susceptibility of people for ill health from CAFO exposures on the basis of age, gender, or genetic makeup. This workgroup expressed the view that international harmonization is needed for analytical methods for exposure assessment of biological agents such as bacterial endotoxin, fungal glucan, and other pathogen-associated molecular patterns. Additionally, they noted that recent advances have identified less invasive approaches for collection of body fluids from which more sensitive biomarkers of response can be measured. They recommended that panel studies be performed among susceptible populations exposed to CAFO emissions, as this approach is most effective for determining responsible agents and disease mechanisms. In terms of science translation to policy, they recommended that best practices for occupational hygiene be promoted for the livestock industry and that exposure standards for organic dust, biological agents, and toxic gases should be promulgated and enforced across the industry.

The Workgroup on Modeling and Monitoring of Emissions from CAFOs noted that the downstream concentrations of airborne effluents from CAFOs are not well understood (Bunton et al. 2006). They recommended establishment of monitoring networks for hydrogen sulfide and ammonia using many low-cost passive monitors and a lesser number of expensive realtime monitors. Some monitors should be located in relatively pristine areas away from livestock operations in order to characterize background levels in rural areas. There is a further need for particulate monitoring accompanied by analysis of adsorbed malodorous vapors and gases, since these appear to travel up to a kilometer from the source. This workgroup found that additional studies should seek to identify links between specific agents ascribed to CAFO emissions and health outcomes in the rural community. In terms of modeling fate and transport from livestock operations, the workgroup found that additional data are needed on emission rates from manure storage tanks or lagoons, land-applied manure, and livestock buildings that are tied to animal inventories and management practices. The workgroup determined that modeling has advanced as a science and should be better utilized for decisions on permitting, siting, and waste management of CAFOs. Further refinements should include models that account for chemical transformation of effluents and models that provide long-term concentration distributions at a regional level.

The Workgroup on Impacts of CAFOs on Water Quality listed several priority research areas, including monitoring of whole watersheds in order to understand the effects of extreme events on ecosystem health, toxicologic assessment of water contaminants from CAFOs, and studies of primary effluents and metabolites in soils, sediments and water (Burkholder et al. 2006). This workgroup recommended surveillance programs for rural private well water in areas at high risk for contamination. They suggested that effective waste and wastewater treatment practices known for managing human wastes, augmented with emerging technologies, should be translated into practice to prevent consumption of emerging contaminants such as veterinary pharmaceuticals (including antibiotics and anabolic hormones). The workgroup identified a need for implementation of best management practices through education and regulation to reduce release of CAFO contaminants into surface waters and aquifers.

The Workgroup on The Potential Role of CAFOs in Infectious Disease Epidemics and Antibiotic Resistance raised concerns about the practice of co-locating swine and poultry facilities and the specter of a global pandemic arising from new strains of avian influenza incubated in swine and transmitted to humans (Gilchrist et al. 2006). They recommended that minimum separation distances should be established and that animals should not be fed tissues, fecal matter, or contaminated water from other animals. This workgroup stated that solid tanks for storage of manure and municipal style waste treatment are necessary to limit microbial contamination of soil and water, prevent access to waterfowl, and limit the spread of disease. The workgroup strongly endorsed phasing out the use of antimicrobial agents as growth promotants in the United States, as is happening in the European Union and was called for by the World Health Organization and dozens of scientific and medical organizations. One complication is a difference between the United States and the European Union animal industries’ interpretation of the terms “growth promoter” and “therapeutic use.” In the United States some routine, nontherapeutic uses of antibiotics are not considered to be growth promotion, whereas in the European Union, they are defined as such. At the time Denmark phased out antibiotic use for animal growth promotion, all remaining antibiotic uses with animals were administered by prescription only. This phase-out resulted in an overall drop in antibiotic use of about 54%. On the other hand, the U.S.-based Animal Health Institute, which respresents pharmaceutical manufacturers, has in the past stated that only about 13% of antibiotic use in U.S. animal production is for growth promotion, and that 87% is for therapeutic use, and almost all U.S. antibiotics used in animal production are available over-the-counter. This differentiation is important, as a phase-out of antibiotics used for growth promotion as defined in the United States would likely result in a much smaller reduction (13%) than the phase-out of growth promotion in Denmark (54%), given that Denmark’s numbers include some antibiotics administered routinely for disease prevention or therapy. The workgroup identified a need to establish national surveillance programs to track the transmission of antimicrobial-resistant organisms from livestock to humans and to identify ecologic reservoirs and impacts. Fingerprinting of antibiotic-resistant bacteria is a necessary component and will allow characterization of changes in resistance profiles over time.

The Workgroup on Community Health and Socioeconomic Issues Surrounding CAFOs considered the impacts of industrialization of livestock production on rural communities in terms of economics, social capital and quality of life (Donham et al. 2006). They recommended comprehensive community health studies comparing physical, mental and social health outcomes, and economic conditions in comparable communities with and without large livestock operations. This workgroup noted that much of the research funding for agriculture is directed toward nonsustainable production and recommended that funds be reoriented to sustainable systems. The workgroup concurred that there is sufficient information on the hazards of CAFOs to communities that a more measured approach to siting and permitting of facilities and waste management is needed and that permits should consider watershed level animal density and dispersion of airshed emissions. Decisions concerning the issuance of permits should also include greater involvement of communities through public hearings and open meetings. The workgroup suggested that permits for manure storage reservoirs should require bonding to ensure that spills will be cleaned up and manure lagoons will be decommissioned rather than abandoned, should the producer become insolvent.

There was general agreement among all workgroups that the industrialization of livestock production over the past three decades has not been accompanied by commensurate modernization of regulations to protect the health of the public, or natural public-trust resources, particularly in the United States. Even though the European Union has made greater strides, there is room for improvements in the control of air and water pollutants from CAFOs in Europe as well as the United States. Expansion of large CAFOs into central and eastern Europe and South America is occurring without attention to lessons learned from health and environmental problems in the United States. and western Europe. Major concerns exist over the role of intensive livestock production in influenza outbreaks and the emergence of antibiotic resistant organisms. Recent attention to these risks among the scientific community, the public, and governments is encouraging.
